# Resveratrol Provides Cardioprotection after Ischemia/reperfusion Injury via Modulation of Antioxidant Enzyme Activities 

**Published:** 2013

**Authors:** Meherzia Mokni, Sonia Hamlaoui, Ines Karkouch, Mohamed Amri, Lamjed Marzouki, Ferid Limam, Ezzedine Aouani

**Affiliations:** a *Neurophysiology Laboratory and Functional Pathology, Department of Biological Sciences, Faculty of Sciences of Tunis, University Campus of Al-manar, Tunisia, Tunis.*; b *Bioactive Substance Laboratory, Biotechnology Centre, Technopolis Borj-Cedria, BP-901, 2050 Hammam-Lif, Tunis. *

**Keywords:** Resveratrol, Heart, Ischemia/reperfusion injury, MDA, Free iron, Catalase, Superoxide dismutase, Peroxidase, Antioxidant enzyme isoforms

## Abstract

In this study, we investigated the cardioprotective effects of resveratrol. Rats were intraperitoneally administered with resveratrol (25 mg/kg bw) or vehicle (ethanol 10%) for 7 days and their heart subjected to ischemia/reperfusion injury. Isolated hearts were langendorff perfused, left ventricular functions as heart rate and developed pressure, as well as, heart antioxidant status were determined.

Data showed that resveratrol improved recovery of post-ischemic ventricular functions when compared to control. Resveratrol also improved myocardial lipoperoxidation, free iron and antioxidant enzyme activities. Resveratrol decreased significantly catalase while it increased peroxidase and superoxide dismutase activities. In this later case, native PAGE analysis of superoxide dismutase isoforms revealed that resveratrol up regulated iron and manganese isoforms. Resveratrol exerted potential cardioprotection partly by its antioxidant properties.

## Introduction

Stroke is a major cause of morbidity and mortality all over the world. Although the pathophysiological mechanisms have not been fully explored, an increase in oxidative stress has often been involved (Manzanero *et al*., 2012). Reactive oxygen species (ROS) induced the oxidation of membrane lipids leading to increased levels of malondialdehyde, a specific biomarker of lipoperoxidation (Michel *et al*., 2008). 

The ischemic and reperfused heart is a good model for the study of heart stroke. The prevention of this stroke using antioxidant enzymes or natural product able to behave as potential inducers of such enzymes (Maksimenko and Vavaev, 2012), is of particular interest. Resveratrol (RVT) is a phytoalexin abundantly found in grapes (Ren and Lien, 1997), exhibiting multiple biological and pharmacological properties including antioxidant and anti-inflammatory effects (Smoliga *et al., *2011). However, the full extent and nature of the cardiovascular effects of RVT particularly its potential actions on the myocardium, should be analysed. We recently demonstrated (Mokni *et al*., 2007a) the ability of RVT to improve hemodynamic parameters of ischemic heart by NO independent way. 

In the present study, we have evaluated the putative involvement of antioxidant enzymes on resveratrol cardioprotective effect. Our results show that pre-treatment with resveratrol efficiently suppresses I/R-induced loss of contractile activity thanks to its antioxidant activity.

## Experimental

*Reagents *Resveratrol was purchased from Selmedica Healthcare (Korea). 2-Thio-barbituric acid (TBA) was purchased from Sigma chemicals Co (Germany). All other chemicals were of analytical grade.

*Animals and treatment*

Male Wistar rats from Pasteur Institute; Tunis (220–240 g) were used in these experiments in accordance with the Ethic Committee of Tunis University for the care and use of animals in conformity with NIH guidelines. They were provided with food and water ad libitum and maintained in animal house at fixed temperature of 22 ± 2°C with a 12 h light–dark cycle. Animals were divided into two groups of 6 animals each. Groupe 1: control injected with 10% ethanol 10% and group 2, resveratrol treated (25 mg/kg bw) daily administered by intraperitoneal injection for 7 days.

*Heart perfusion and hemodynamic assessment*

24 h after the last injection, rats were anesthetized with 0.5 mL urethane (40 mg/mL) and heparinized (1.1 U/L). Hearts were rapidly isolated and arrested in ice-cold perfusion krebs-Henseleit (KH) buffer. The aorta was cannulated and the heart was Langendorff-perfused at a constant pressure of 70 mmHg in continuously gassed prewarmed KH buffer. Hearts were subjected to stabilization for 10 min before a global ischemia period of 45 min followed by 10 min reperfusion. Hemodynamic parameters were monitored as described previously (Mokni *et al*., 2007 b). At the end of ischemia/reperfusion (I/R) damage, hearts were weighed, homogenized in phosphate buffer saline pH 7.4 with an ultrathurax T25 homogenisator, centrifuged (10 min at 10 000 g, 4°C) and supernatant used for measurement of free iron level, malondialdehyde (MDA) and antioxidant enzyme activities. 

*Free iron determination*

Myocardial free iron was determined according to Leardi *et al*. (1998) using a commercially available kit from Biomaghreb (Ariana, Tunisia). Briefly, at acidic pH 4.8 all Fe^3+ ^released from transferrine were reduced by ascorbic acid into Fe^2+^, which constitutes with ferrozine a purple colourful complex measurable at 560 nm. Heart extract was added to 250 μl of reaction mixture containing ascorbic acid (5 g/L) and ferrozin (40 mM), and incubation was performed at 37°C for 10 min.

*Lipoperoxidation determination*

Lipid peroxidation was determined by malondialdehyde (MDA) measurement according to the double heating method (Draper and Hadley, 1990). Briefly, aliquot from heart homogenate was mixed with BHT-TCA solution containing 1% BHT (m/v) dissolved in 20% TCA (m/v) and centrifuged at 1000 g for 5 min at 4°C. The supernatant was blended with 0.5N HCl, and 120 mM TBA in 26 mM Tris, and then heated at 80°C for 10min. After cooling, absorbance of the resulting chromophore was determined at 532 nm using a Bio-Rad UV-visible spectrophotometer. MDA levels were determined by using an extinction coefficient for MDA-TBA complex of 1.56 10^5^ M^-1^cm^-1^. 

*Antioxidant enzyme activity assays*

All spectrophotometric analyses of antioxidant enzyme activities were performed with a SmartSpec 3000 Bio-Rad UV-visible spectrophotometer (Germany).

Catalase (CAT) activity was assayed by measuring the initial rate of H_2_O_2_ disappearance at 240 nm (Aebi, 1984). The reaction mixture contained 33 mM H_2_O_2_ in 50 mM phosphate buffer (pH 7.0) and heart extract. CAT activity was calculated using an extinction coefficient of 40 mM-1cm^-1^ for H_2_O_2_.

Peroxidase (POD) activity was measured at 25°C using guaiacol as the hydrogen donor. The reaction mixture contained 9 mM guaiacol, 19 mM H_2_O_2_ in 50 mM phosphate buffer pH 7 and 50 μL of heart homogenate extract in a final volume of 1 mL. The reaction was initiated by the addition of H_2_O_2_ and monitored by measuring the increase in absorbance at 470 nm every 30 sec for 3 min. POD activity was expressed as nmol of guaiacol oxidized per min and calculated using a molecular extinction coefficient of 26.2 mM^-1^ (Chance and Maehly, 1955).

Superoxide dismutase (SOD) activity was determined by using a modified epinephrine assay (Misra and Fridovich, 1972). At alkaline pH, superoxide anion (O_2_^-^) causes the auto-oxidation of epinephrine to adenochrome. One unit of SOD is defined as the amount of extract that inhibits the rate of adenochrome formation by 50%. Heart homogenate was added to a 2 mL reaction mixture containing 10 μl bovine catalase (0.4 U/μL), 20 μL epinephrine (5 mg/ml) and 62.5 mM sodium carbonate-sodium bicarbonate buffer (pH 10.2). Changes in absorbance were recorded at 480 nm. 

*Analysis of antioxidant isoenzyme activities by Polyacrylamide gel electrophoresis (PAGE) *Antioxidant SOD isoforms were analyzed by native PAGE. Acidic isoforms were resolved in 4% stacking and 10% separating gel according to Davis (1964). Basic isoforms were resolved in 4% stacking and 10% separating gel according to Reisfeld *et al. *(1962) with slight modifications. Briefly, gels were prepared in 25 mM acetic acid/KOH pH 6 buffer system and migration was conducted in 40 mM glycine/HCl pH 4 at 120 V for 3 h at 4°C. Electrophoresis were carried out toward the cathode allowing only neutral and basic proteins separation. 

SOD activity was revealed after incubation of gels in 2.5 mM nitroblue tetrazolium (NBT) solution for 30 min and stained with 28 mM Temed and 28 μM riboflavin in 50 mM potassium phosphate buffer pH 7.8 (Beauchamp and Fridovich, 1971). Characterization of SOD isoforms was performed using KCN (5 mM) which inhibited Cu/Zn-SOD or with H_2_O_2_ (5 mM) affecting both Cu/Zn-SOD and Fe-SOD. Whereas, Mn-SOD was insensitive to both inhibitors (Loukhili *et al., *1999). 

CAT isoenzymes were separated by native or SDS 7% PAGE according to Davis (1964). CAT activity was visualized after soaking the gel in 3.27 mM H2O2 for 30 min, washing in double distilled water and stained with a mixture of 1% (w/v) potassium ferricyanide and 1% (w/v) ferric chloride (Woodburry *et al*., 1971). 

POD isoforms were analyzed by native 10% PAGE according to Davis (1964). POD activity was revealed after incubating the gel for 5 min in 100 mM sodium acetate buffer pH 5 containing 2.17 mM benzidine, 1% guaiacol and 0.5% H2O2 according to Lee *et al*. (2001).

*Densitometry*

Densitometric values of the protein band activities were evaluated using image J software and expressed in arbitrary units (AU).

*Statistical analysis*

Data were analyzed by unpaired Student’s t-tests or one-way analysis of variance (ANOVA) and expressed as means ± standard error of the mean (SEM). All statistical tests were 2-tailed, and results with a p-value < 0.05 were considered significant. 

## Results

**Figure 1:**
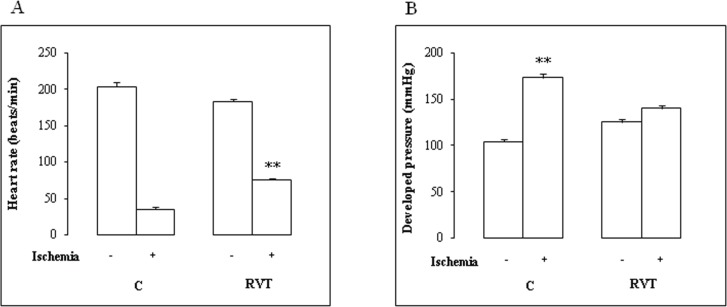
Effect of RVT on heart rate variations of isolated heart subjected to I/R injury. Heart rate was recorded before (-) and after (+) .ischemia in control (C) and RVT treated animals. Results are expressed as mean ± SEM (n=6). **p < 0.01 vs C

Data of heart rate and developed pressure before and after ischemia are illustrated in Figure1. There was no difference in baseline function between control and RVT group. On reperfusion, RVT increased the absolute values of heart rate (Figure 1A) (+ 213%). Thus, a one-week RVT treatment significantly improved contractile function of perfused hearts (Figure 1B). 

**Figure 2:**
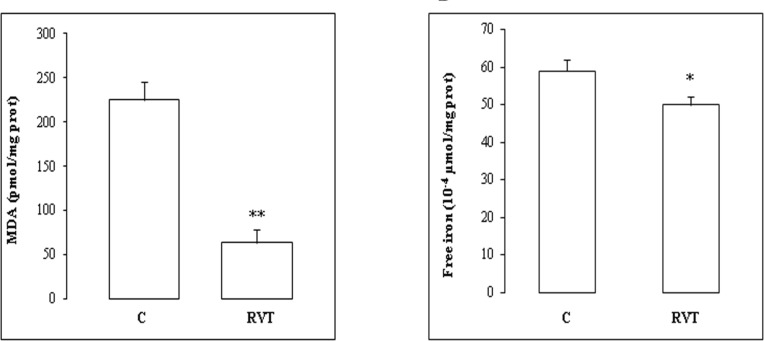
Antioxidant effect of resveratrol. After I/R insult, isolated hearts from C or RVT treated animals were used for MDA (Figure 2A) and free iron (Figure 2B) determinations. Results are expressed as means ± SEM (n=6). *p < 0.05 vs C. **p < 0.01 vs C

We then determined the level of myocardial MDA and free iron (Figure 2). RVT treatment (without ischemia) dropped heart lipid peroxidation by 72% (Figure 2A) and free iron by 21% (Figure 2B). 

**Figure 3:**
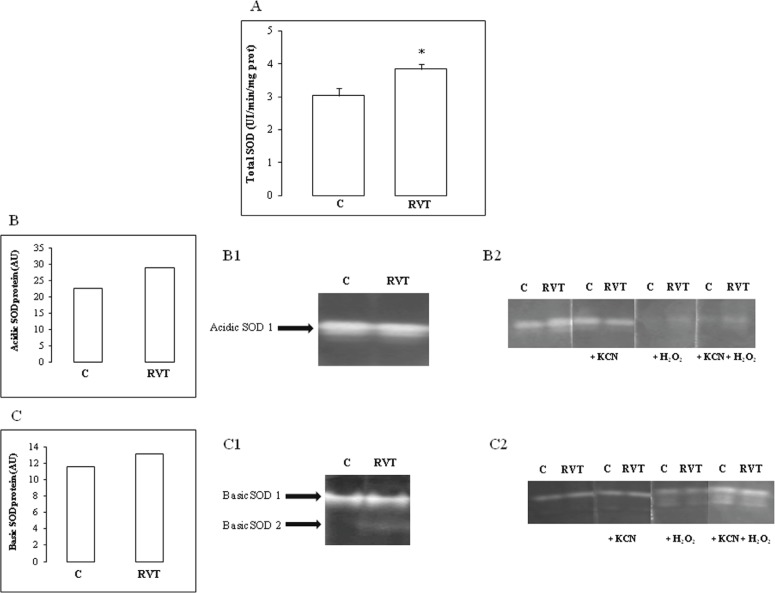
Effect of resveratrol on SOD activity and isoenzyme pattern. Total SOD activity measurement (Figure 3A). A representative pattern of a major acidic SOD isoenzyme called SOD A1 (Figure 3B) which is up-regulated by RVT and identified as an Fe-SOD (Figure 3B1; 3B2). A representative pattern of a major basic SOD isoenzyme called SOD B1 (Figure 3C) which is up-regulated by RVT and identified as a Mn-SOD (Figure 3C1; 3C2). Results are expressed as means ± SEM (n=6). *p < 0.05 vs C

RVT treatment increased total SOD activity in heart homogenates (Figure 3A). Characterization of SOD isoforms under native acidic PAGE revealed that RVT increased Fe-SOD (Figure 3B, B1, B2) whereas under native basic PAGE, the polyphenol increased the Mn isoform (Figure 3C, C1, C2). RVT had no significant effect on Cu/Zn-SOD. 

**Figure 4:**
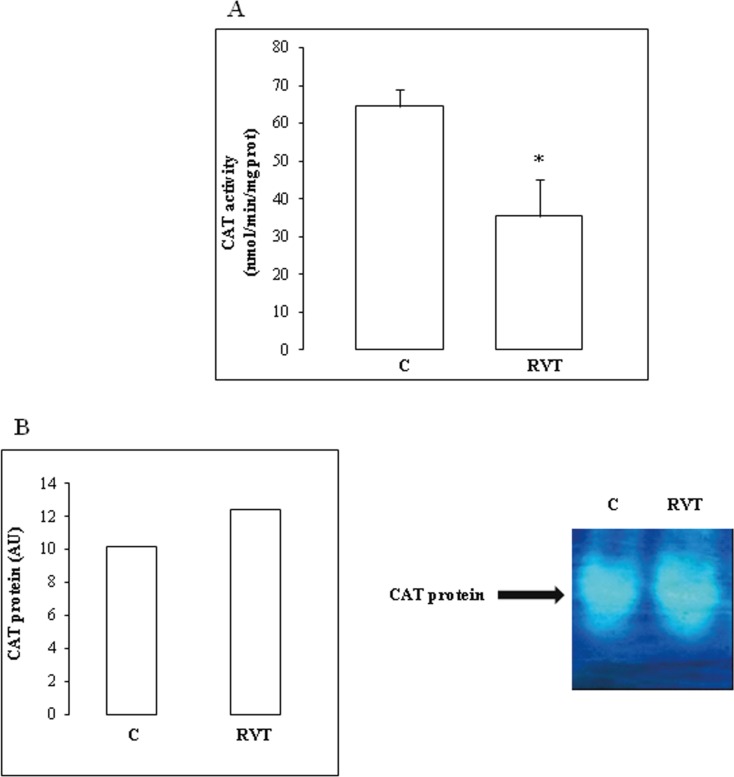
Effect of resveratrol on CAT activity and isoenzyme pattern. RVT inhibited CAT activity (Figure 4A) but had no effect on isoform abundance (Figure 4B). Values are mean ± SEM (n=6). *p < 0.05 vs C

Furthermore, RVT decreased total myocardial CAT activity by 45%) (Figure 4A). PAGE analysis under native or denaturing conditions showed that RVT had no significant effect on CAT protein band intensity (Figure 4B). 

**Figure 5:**
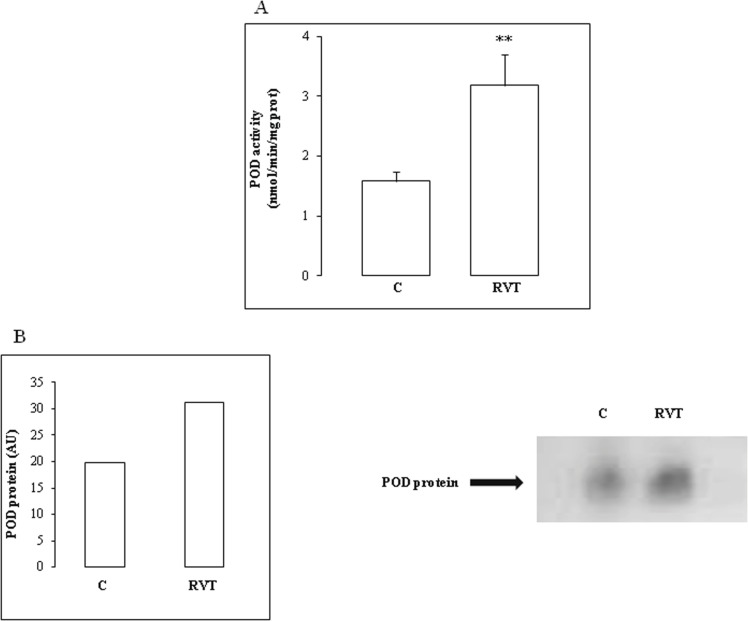
Effect of resveratrol on total POD activity and isoenzyme pattern. POD activity (Figure 5A) and isoform abundance (Figure 5B) are both increased by RVT treatment. Values are mean ± SEM (n=6). **p < 0.01 vs C

RVT induced a significant rise in POD activity by 100% and POD isoform abundance by 50% (Figure 5B). 

## Discussion and Conclusion

Our previous work (Mokni *et al., *2007a), the findings of Hung *et al*. (2000) demonstrated the efficiency of RVT in the prevention and improvement of post-ischemic ventricular functional recovery (de Lorgeril *et al*., 2003) and in the management of cardiovascular dysfunction. Our data confirmed that RVT exerted beneficial effects on ischemic heart partly by its antioxidant property. In fact, RVT reduced myocardial MDA level and increased SOD and POD activities. It is noteworthy that among SODs, Fe and Mn isoforms were selectively up-regulated whereas Cu/Zn- SOD was unchanged. Cu/Zn-SOD is believed to play an important physiological role in liver homeostasis (Uchiyama *et al., *2006) whereas Fe-SOD has a more prominent role in iron overload-induced cardiac disorders (unpublished data). Accordingly, we have recently demonstrated that RVT up-regulated SOD in the brain of healthy rat and that Fe and Mn were the predominant isoforms when compared to Cu/Zn-SOD (Mokni *et al*., 2007b). In addition, RVT also enhanced POD activity in the heart, thereby confirming previous data found in the brain (Mokni *et al*., 2007b) and kidney (Sebai *et al*., 2008) and RVT also appeared as acting independently from its classical and well-documented antioxidant effect. 

Besides, RVT decreased myocardial free iron level. Free iron is a well known inducer of iron-catalysed Fenton reaction giving rise to harmful ROS as hydroxyl radicals. The deleterious effect of iron accumulation in the heart is well documented in diseases of systemic iron overload (Aryeh *et al*., 2012). In this context, it is tempting to speculate that RVT acted on L-type voltage-dependent Ca^2+ ^channels to decrease iron overload. Interestingly, such channels have recently been shown to be an important means for iron entry into cardiomyocyte. Oudit *et al. *(2003) attenuated myocardial iron accumulation and oxidative stress in excitable cells after blocking the Ca^2+^ channel using verapamil. Consequently, our data which emphasized a direct role of RVT on iron channels controlling the excitability of the myocardium, are consistent with the use of RVT as a safe and cardioprotective agent in the treatment of severe cardiac dysfunction as arrhythmia (Zhang *et al*., 2006; Merle *et al*., 2007). However, as another alternative, RVT could modulate iron regulatory hormone as hepcidin. The autocrine formation of this hormone in the heart has recently been demonstrated (Merle *et al*., 2007), but its exact role in failing heart is uncertain (Isoda *et al*., 2009). A third hypothesis could be the iron chelating property of RVT, as described for another polyphenol, namely, quercetin (Leopoldini *et al*., 2006). Iron chelators are very useful in iron depletion-mediated cell cycle arrest, apoptosis and anticancer activity (Dong and Richardson, 2007; Gérard *et al., *2012). 

On the other hand, by down-regulating CAT activity, RVT also appeared as acting independently from its classical antioxidant way. At this level, a specific point must be emphasized concerning the effect of RVT on CAT activity and protein abundance. RVT depressed CAT activity but had no significant effect on protein abundance. One can speculate about post-translational modifications of antioxidant enzymes. Theses modifications could induce a loss of function but a gain of radical generating ability which are consistent with the recently described and unexpected pro-oxidant effect of CAT (Heck *et al*., 2003). Thus, Mn-SOD from Listeria Monocytogenes can be down-regulated by phosphorylation in the host cell (Archambaud *et al*., 2006). Moreover, in mammalian cells, an important link between phosphorylation of antioxidant protein as peroxiredoxin I and cell cycle progression has been described (Chang *et al*., 2002). The effect of RVT on cardiac CAT may have some importance with regards to the hypertrophic stimuli, in which insulin was shown to induce cardiac hypertrophy by down-regulating CAT (Murtaza *et al*., 2008). The effect of RVT described here *i.e*. the improvement of cardiac dysfunction seemed to convey by the same pathway and needed further investigation especially concerning the involvement of ROS as a downstream mediator of RVT. At a first glance, it seems this is not the case if we consider the RVT effect on MDA level. Further work aiming to determine the putative effect of RVT on ROS species as H_2_O_2_ is in progress. Preliminary data (not shown) seems to indicate a link between RVT induced variations in H_2_O_2_, CAT and free iron levels in rat myocardium. 

We have recently demonstrated opposite effects of RVT as either pro or antioxidant in chronobiological investigation, *i.e*., when the polyphenol was administered at different times in the 24h scale (Gadacha *et al*., 2009). Furthermore, our data had shown opposit results in comparison with previous work. Indeed, we have shown (Mokni *et al*., 2007b) that RVT afforded neuroprotection by up-regulating CAT activity, whereas in the present work RVT exhibited cardioprotective effects and down-regulated CAT activity. In a comparative study of RVT effect between various organs as kidney, liver, brain and heart, we confirmed these dicrepancies (data not shown). In our opinion, such discrepancies might be interpreted by the differential time and dose-related effects of RVT but also versus the specific threshold each organ displayed towards RVT. These discrepancies are probably linked to the presence of polyphenol specific membrane receptors which eventually lead to pro or antioxidant effect. 
